# Identification of Recurrence Related microRNAs in Hepatocellular Carcinoma after Surgical Resection

**DOI:** 10.3390/ijms14011105

**Published:** 2013-01-08

**Authors:** Zhen Yang, Ruoyu Miao, Guangbing Li, Yan Wu, Simon C. Robson, Xiaobo Yang, Yi Zhao, Haitao Zhao, Yang Zhong

**Affiliations:** 1 Department of Liver Surgery, Peking Union Medical College Hospital, Chinese Academy of Medical Sciences and Peking Union Medical College (CAMS & PUMC), Beijing 100730, China; E-Mails: zhenyang@fudan.edu.cn (Z.Y.); lgb002@126.com (G.L.); y110403606@126.com (X.Y.); 2 Institute of Biomedical Sciences, Fudan University, Shanghai 200433, China; 3 School of Life Science, Fudan University, Shanghai 200032, China; 4 Bioinformatics Research Group, Center for Advanced Computing Technology Research, Institute of Computing Technology, Chinese Academy of Sciences, Beijing 100190, China; E-Mails: miaoruoyu@ict.ac.cn (R.M.); biozy@ict.ac.cn (Y.Z.); 5 Department of Liver Transplantation and Hepatobiliary Surgery, Provincial Hospital Affiliated to Shandong University, Jinan, Shandong 250021, China; 6 Liver Center and Transplant Institute, Department of Medicine, Beth Israel Deaconess Medical Center, Harvard University, Boston, MA 02215,USA; E-Mails: ywu@bidmc.harvard.edu (Y.W.); srobson@bidmc.harvard.edu (S.C.R.); 7 Institute of Biodiversity Science and Geobiology, Tibet University, Lhasa 850000, China

**Keywords:** microRNA, expression, hepatocellular carcinoma, recurrence

## Abstract

Hepatocellular carcinoma (HCC) is one of the most aggressive human cancers with a high frequency of post-surgical recurrence. It is very critical to diagnose HCC recurrence at an early stage for a better therapeutic treatment. In this study, we examined the microRNA (miRNA) expression profiling in tumor tissues obtained from early and late recurrent HCC patients post-resection, using a microarray assay. A total of 32 miRNAs were identified to be differentially expressed during the progression of recurrence. Among these, 16 miRNAs were upregulated and 16 were downregulated. In addition, this miRNA expression signature was further validated by quantitative real-time polymerase chain reaction (qRT-PCR) analysis. Moreover, functional annotation of predicted target genes of these recurrent HCC-related miRNAs indicates that multiple biological pathways (*i.e.*, focal adhesion pathway, cancer-related pathways and mitogen-activated protein kinase (MAPK) signaling) that are all critical for cancer development and progression, may participate in the recurrence of HCC. Our data suggest potential molecular mechanisms underpinning miRNA-controlled HCC recurrence, and support the notion that miRNA expression signature and miRNA-based therapy can be useful tools for a better diagnosis and treatment stratification of this disease.

## 1. Introduction

Hepatocellular carcinoma (HCC), one of the most highly aggressive malignancies of the liver, is the third leading cause of cancer-related mortality. It was reported that, worldwide, more than 500,000 people were newly diagnosed with HCC causing 600,000 deaths annually. This number is still increasing in many countries [1,2]. HCC is also extremely difficult to manage. To date, surgical resection and orthotopic liver transplantation remain the best curative options for treatment of HCC. However, only 5%~15% of HCC patients are currently eligible for surgical intervention based on the evaluation of their liver function, and the vast majority of HCC patients have unresectable tumors [3]. Moreover, the postoperative five-year survival rate is only 30%~40%, as a result of the high rate of metastasis and recurrence in patients who have had resection [4]. As such, it is urgently required to identify novel biomarkers and high-risk factors, as well as to elucidate the molecular mechanisms underlying HCC recurrence.

Recently, several useful biomarkers inclusive of microRNAs (miRNAs) have been identified to predict the prognosis, metastasis and overall patient survival of HCC [5]. miRNAs are small noncoding RNAs that post-transcriptionally regulate gene expression [6,7]. They are processed from a hairpin precursor of 60–80 nt long, which is originated from a much longer messenger RNA-like transcript that is mainly transcribed by RNA polymerase II [8]. The major function of miRNAs is to induce translational inactivation or degradation by binding to the 3′-untranslated region (3′-UTR) of target mRNAs in a sequence-specific manner [9]. Up to date, more than 1000 miRNA genes have been identified in the human genome (miRBase release 17.0) [10], in which one third of the genes are thought to be modulated by miRNAs [11]. These miRNAs participate in many key cellular processes including proliferation, apoptosis and metastasis [12]. Deregulation of miRNAs has been linked to many diseases, especially cancer. So far, a number of liver-enriched miRNAs in humans have been identified, and their roles as etiologic factors in HCC have also been suggested [13].

Chronic infection with hepatitis B virus (HBV) or hepatitis C virus (HCV) is considered the leading cause of the malignant transformation of HCC. Several miRNAs have been shown to be deregulated in HBV-related HCC progression. For example, miR-29a could facilitate HBV protein HBx-induced hepatoma cell migration by modulating phosphatase and tensin homolog (PTEN) and its downstream component protein kinase B (Akt) [14]. MiR-196 was found to inhibit HCV expression and repress HCV-induced oxidative damage [15]. Evidence has also suggested that miRNAs are widely involved in carcinogenesis, differentiation and metastasis of HCC. Murakam *et al.* systematically analyzed miRNA expression profiles in biopsies from patients with HBV, HCV, cirrhosis, and HCC using the adjacent normal tissues as control, and demonstrated that levels of miR-18, pre-miR-18 and miR-224 are elevated in HCC, whereas levels of miR-199a*, miR-195, miR-199, miR-200a and miR-125a are decreased. Additionally, they also linked six miRNAs to the differentiation degree of HCC [16]. Budhu *et al.* evaluated miRNA expression patterns from 241 HCC patients and noted that 20 miRNAs are associated with metastasis [17]. Recent advances in high-throughput methods for analysis of miRNA expression profiling in HCC provide more informative and accurate molecular tools for HCC prognosis. Using miRNAs as drug targets for molecular therapy has also implicated their clinical potential in treating HCC [18].

In this study, we examined the miRNA expression profiling from HCC patients who have had surgical resection to identify recurrence-related miRNAs, using a miRNA array. We found that 32 miRNAs are differentially expressed in tumors of HCC patients with different postoperative survival. Functional annotation of the miRNA target genes was further performed to explore the putative links between these miRNAs and HCC recurrence. We demonstrated that signaling pathways involved in many fundamental biological processes are linked to the recurrence of HCC, namely focal adhesion pathway, cancer-related and MAPK signaling, *etc.* Our data suggest the potential for mechanistic insights and the utility of miRNA-based drugs as novel therapeutic strategies in preventing recurrence of HCC.

## 2. Results and Discussion

### 2.1. Patients and Samples

The patients enrolled in the study were randomly selected. HCC biopsies were obtained from recurrent patients who underwent surgical resection between 2003 and 2010 at the Department of Liver Surgery, Peking Union Medical College Hospital (Chinese Academy of Medical Sciences and Peking Union Medical College, Beijing, China). Among 15 samples analyzed, eight are from patients with early recurrence (less than six months) and seven are late recurrent (more than six months). The median recurrence-free period was 2.3 months for patients with early recurrence and 44.8 months for those with late recurrence. All patients had cirrhosis and micro-vascular invasion, and 80% had a serum α-fetoprotein level > 20 ng/mL. There was no difference in other clinicopathological features between these two groups of patients (Table 1).

### 2.2. Identification of Recurrence-Related miRNAs in HCC Samples

**Figure 1:**
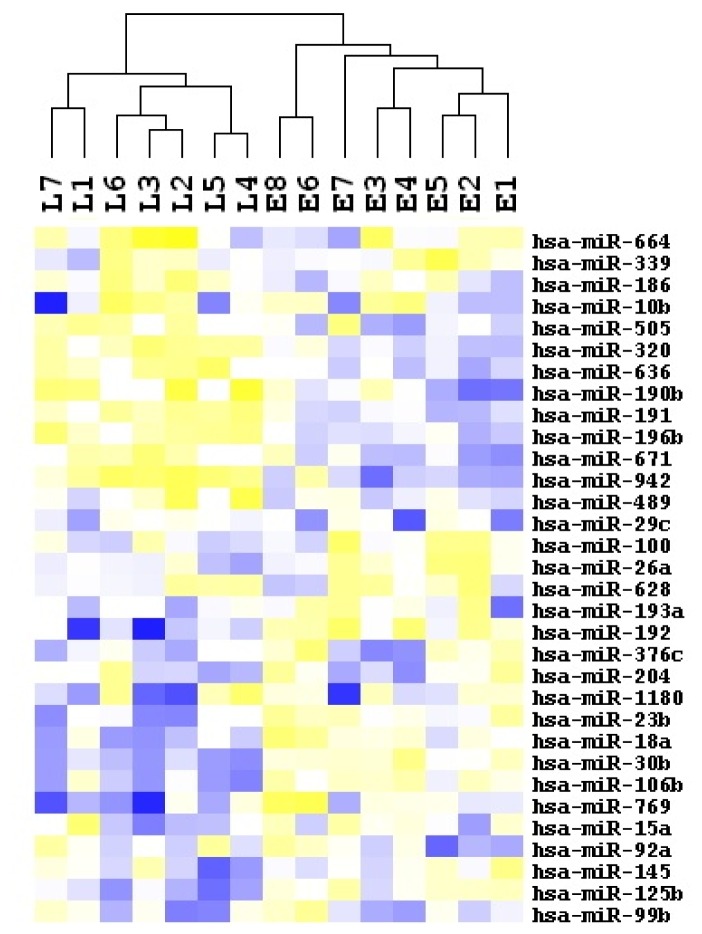
Hierarchical clustering of 32 miRNAs whose expressions were significantly altered in late recurrent HCC samples in contrast with early recurrent HCC samples.

Fifteen human HCC samples were analyzed for high-throughput miRNA expression profiling using a state-of-art microarray method. First, 240 miRNAs in total were obtained after eliminating the ones that are not expressed in most of the cohorts. Next, by comparing the relative expression levels of miRNAs in HCC patients with early recurrence to those with late recurrence, we noted that 32 miRNAs were differentially expressed, with 16 being upregulated and 16 downregulated in late recurrent samples (Table 2). Specifically, upregulation of hsa-miR-636 (30 fold change) and downregulation of hsa-miR-145 (23 fold change) are the most significant changes, as compared with early recurrent samples. Furthermore, unsupervised hierarchical clustering analysis of these differentially expressed miRNAs revealed distinct expression patterns in the two different stages of HCC recurrence (Figure 1), suggesting the potential of expression profiles of these 32 miRNAs to distinguish early recurrent HCC from late recurrent HCC.

### 2.3. Quantitative Real-Time PCR Validation of miRNA Expression

**Figure 2:**
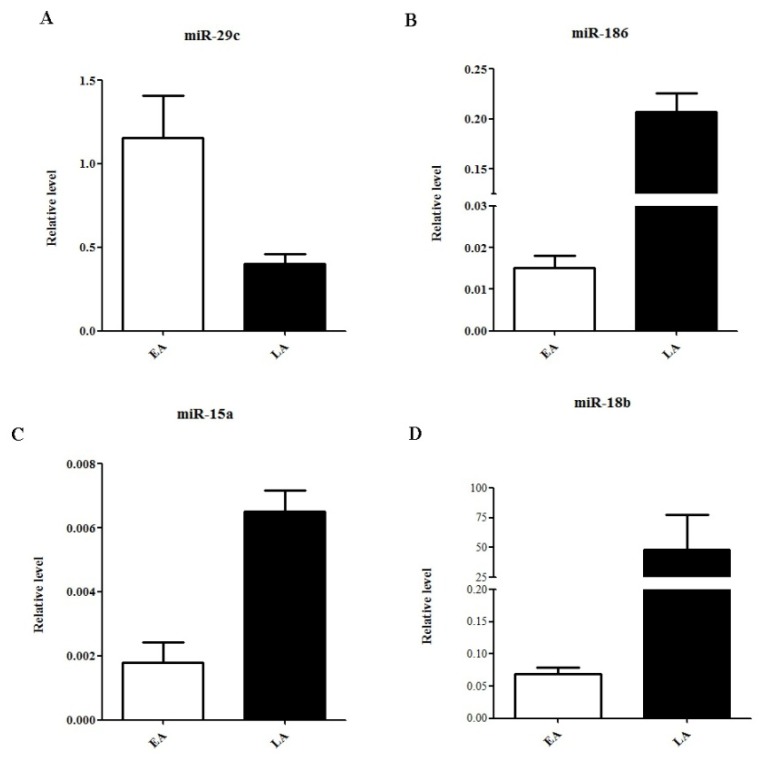
qRT-PCR validation of four select miRNAs. Relative expression of miR-29c (**A**), miR-186 (**B**), miR-15a (**C**), and miR-18b (**D**) in early (EA) and late (LA) recurrent HCC samples. Data are normalized to levels of β-actin. *p*-Value < 0.05 in all miRNAs.

To validate our microassay findings above, we then selected four miRNAs including one downregulated (miR-29c) and three upregulated (miR-186, miR-15a and miR-18b) for qRT-PCR analysis. As shown in Figure 2, the respective levels of miR-29c in early *vs.* late recurrent HCC samples were 1.15 ± 0.25 *vs.* 0.40 ± 0.06 (*p*-value = 0.007). In parallel, levels of miR-186 were 0.02 ± 0.00 *vs.* 0.2 ± 0.02 (*p*-value < 0.05), miR-15a were 0.002 ± 0.001 *vs.* 0.007 ± 0.001 (*p*-value < 0.05), and miR-18b were 0.07 ± 0.01 *vs.* 48.56 ± 28.74 (*p*-value < 0.05). qRT-PCR results completely recapitulated the alteration patterns of all these four select miRNAs in miRNA profiling analysis (Table 2 and Figure 1), confirming a good correspondence between these two analytic platforms.

### 2.4. Gene Ontology (GO) and Kyoto Encyclopedia of Genes and Genomes (KEGG) Pathway Analyses of Predicted miRNA Targets

To gain an overview of miRNA and its targets that are involved in the recurrence of HCC, we predicted the targets of these differentially expressed miRNAs using TargetScan [19] and PITA [20]. In total, 2091 protein-coding genes were predicted to be modulated by the miRNAs identified above. To name a few, PCDHAC1, SATB2, DAG1, CNOT6 and RAP2C that are all critical genes in cancers were demonstrated to have the highest connectivity in the miRNA regulatory network, suggesting that dysregulation of these genes could be of functional importance for the tumorigenic processes.

**Figure 3:**
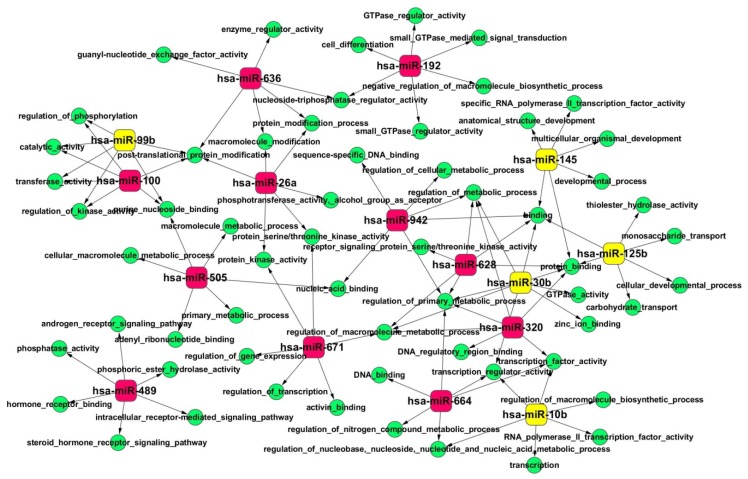
MicroRNA-GO-Network based on the interactions between miRNAs and their gene targets. Red nodes represent the top upregulated miRNAs and yellow nodes are top downregulated, whereas the green nodes are enriched GO terms.

Next, to further explore the functions of these miRNAs in HCC, we selected miRNAs with a fold change >5, namely hsa-miR-636, hsa-miR-671, hsa-miR-489, hsa-miR-26a, hsa-miR-320, hsa-miR-628, hsa-miR-505, hsa-miR-100, hsa-miR-664, hsa-miR-942, hsa-miR-192, hsa-miR-99b, hsa-miR-125b, hsa-miR-10b, hsa-miR-30b, and hsa-miR-145, for GO (Gene Ontology) enrichment analysis [21] of their target genes using the web-based software WebGestalt 2.0 [22]. This method provides a rapid analytic approach categorizing large amounts of genes into functionally related groups to thereby facilitate the uncovering of the biological content captured by transcriptomic profiling. In addition, the hyper-geometric test that is used to classify the GO category and Multiple Test Adjustment of BH was employed here to correct the *p*-value. Based on the predicted interactions of miRNAs with their target genes, the miRNA-GO Network was built (Figure 3). Notably, in the terms of molecular function, significant overrepresentation of GO terms of protein kinase activity, regulation of gene expression, ATP binding, adenyl nucleotide binding, chromatin binding, *etc*, were observed (Figure 3). In parallel, the GO terms of transcription regulation, macromolecule metabolic process, and cell differentiation were identified in the terms of biological process (Figure 3).

Furthermore, to unveil the biological pathways that are altered during HCC recurrence, we also performed the Kyoto Encyclopedia of Genes and Genomes (KEGG) [23] pathway enrichment analysis for the predicted target genes. As demonstrated in Table 3, the top canonical pathways identified here, including focal adhesion signaling, MAPK signaling, and cancer-related pathways, have all been previously linked to tumor development, progression and recurrence [24].

### 2.5. Discussion

Recurrence is the leading cause of the poor outcome of HCC after resection. This gives patients an enormous physiological and psychological burden. However, the cause of HCC recurrence is still not clear. A better understanding of the pathogenesis of HCC could be of paramount importance for drug development and treatment of this disease. Recently, gene expression profiling analysis has been shown to be a useful tool to investigate the pathophysiology of complex genetic tracts, such as cancer [25,26]. It has been speculated that deregulation of a large number of miRNAs, which results in aberrant expression of target genes involving many critical cellular pathways, can be associated with cancer progression.

As candidate biomarkers, differentially expressed miRNAs have been suggested to be useful for diagnosis, treatment, and prognosis of human cancers inclusive of HCC [27–29]. For example, miRNA expression patterns were found to be significantly different in HCC samples, viz. 15 miRNAs exhibited higher expression and one miRNA had lower expression, in contrast to non-tumorous samples [30]. In another independent study using a bead-based expression profiling assay performed on 20 matched HCC and adjacent non-tumorous tissues, 12 upregulated and 19 downregulated miRNAs were found to be associated with HCC [31]. Based on the miRNA expression data, classification of HCC samples achieved an overall prediction accuracy of 97% [16]. These findings have dramatically expanded our knowledge of pathophysiology of HCC.

However, studies focusing on the miRNA expression profiling analysis of HCC patients with different stages of recurrence are somewhat limited as of yet. In this study, miRNA expression profiling analysis was performed on biopsies from HCC patients with well-defined early and late recurrence. We noted that miRNA expression patterns of early recurrent HCC are distinct from those of late recurrent samples. A total of 32 differentially expressed miRNAs (16 upregulated and 16 downregulated) could successfully classify different types of recurrence, implicating the correlation of miRNA expression signature with HCC recurrence.

Indeed, some of these miRNAs have been previously linked to cancer. Specifically, miR-26a was shown to be downregulated in colorectal cancer [32] and cutaneous squamous cell carcinoma [33]. Two nucleotide excision repair (NER) factors, ERCC3 and ERCC4, have been demonstrated to be modulated by miR-192 and overexpression of miR-192 significantly inhibited cellular NER ultimately leading to HCC [34]. MiR-15 was the first miRNA identified participating in B-cell chronic lymphocytic leukemia (B-CLL). Hsa-miR-18 and hsa-miR-125 were upregulated whereas hsa-miR-29c was downregulated in basal cell carcinoma [35]. In our study, although the association of expression of other miRNAs with diseases needs to be further elucidated, we suggest potential biomarkers for identification and classification of recurrent HCC at different stages.

Moreover, we predicted the miRNA targets and utilized GO and KEGG pathway enrichment analyses to further interpret their biological functions. GO analysis of miRNA targets here indicated that miRNAs play critical roles in the regulation of gene expression, metabolic processes, cell-cell adhesion and migration, and apoptosis. In parallel, the top overrepresented pathways identified in the KEGG pathway enrichment analysis include many previously reported cancer-related pathways, e.g., focal adhesion and MAPK signaling.

In summary, our data provide evidence for the underlying biological processes involved in HCC recurrence. Further characterization of pathogenetic roles of specific miRNAs in the recurrence of HCC and deciphering miRNA-controlled signaling regulatory network may help to evaluate and stratify HCC patients for an optimal personalized therapy.

## 3. Experimental Section

### 3.1. Sample Preparation and RNA Extraction

Tumor samples were obtained from HCC patients after hepatic resection and were immediately snap-frozen in liquid nitrogen. MiRNAs were extracted from samples using mirVana™ miRNA Isolation Kit (Ambion, Austin, TX, USA). Concentration of miRNA was determined by NanoDrop 2000 spectrophotometers (Thermo Scientific, Waltham, MA, USA). Genomic DNA was removed from RNAs with RNase-free DNase I (Life Technologies, Inc., Gaithersburg, MD, USA) according to the manufacturer’s instructions. Quality of RNAs was assessed by electrophoresis in ethidium bromide-stained 5% agarose/formaldehyde/MOPS (3-(*N*-Morpholino) propanesulfonic acid) gels and viewed under UV light. The selection criteria of RNA samples were that peaks of the 18 s and 28 s ribosomal RNAs were at least two times higher than other peaks. RNA samples were stored at −80 °C for subsequent analyses.

### 3.2. MicroRNA Expression Profiling

MiRNA array was performed on the 7900HT Fast Real-Time PCR System (Applied Biosystems, Foster, CA, USA) using TaqMan Array Human MicroRNA A+B Cards Set v3.0 (Applied Biosystems, Foster, CA, USA). Briefly, megaplex pools were prepared from 100 ng of total miRNA derived from each sample with TaqMan MicroRNA Reverse Transcription Kit using Megaplex™ RT Primers (Applied Biosystems, Foster, CA, USA). PCR reaction were performed using Taqman Universal PCR Master Mix (Applied Biosystems, Foster, CA, USA) with Megaplex™ RT products as templates. The PCR reaction mixtures were then loaded on Low Density Array Plate (Applied Biosystems, Foster, CA, USA) for miRNA expression profiling. Assay results were collected and analyzed using SDS 2.2.2 software.

### 3.3. Statistical Analyses

The Significance Analysis of Microarrays (SAM) method was used for detection of the differentially expressed miRNAs between early and late recurrent HCC patients [36]. SAM identifies statistically differentially expressed genes by carrying out gene specific *t*-statistics and a “relative difference” score for each gene. The D value was defined as the average expression change from different expression states to the standard deviation of measurements for that gene. Random permutation of the measurement was performed to estimate the false discovery rate (FDR). Genes exhibiting a fold change of at least +2 with a FDR less than 0.05 were selected as the significantly differentially expressed genes (DEGs). Unsupervised hierarchical clustering analysis of differentially expressed miRNAs was performed by Cluster and Treeview.

### 3.4. Reverse Transcription and Quantitative RT-PCR Validation

cDNA was reverse transcribed from total miRNA with the TaqMan^®^ MicroRNA Reverse Transcription Kit (Applied Biosystems, Foster, CA, USA) using small RNA-specific (miR-15a, miR-18b, miR-29c and miR-186), stem-loop RT primers supplied by Applied Biosystems. First strand cDNA was synthesized from 1 ng of total miRNA in a 15 μL reaction system. 1.33 μL of cDNA was used as template for qRT-PCR validation. qRT-PCR assays of mature miR-15a, miR-18b, miR-29c and miR-186 were performed on StepOnePlus™ Real-Time PCR System (Applied Biosystems, Foster, CA, USA) using the TaqMan^®^ Small RNA Assays (Applied Biosystems, Foster, CA, USA). U6 was used as an internal control. All reactions were performed in triplicates. All primers for qRT-PCR were validated and supplied by Applied Biosystems. Assay results were collected and analyzed using StepOne Software v2.2, and presented as the C_T_ value.

### 3.5. miRNA Target Prediction and Functional Analysis

The predicted targets of miRNAs were obtained from TargetScan database [37], and PITA database [38]. The intersections of results obtained from these different software programs were regarded as the reliable target genes. The Gene Ontology enrichment analysis was performed to investigate the main function of target genes using the web-based software WebGestalt 2.0. Hyper-geometric test was used to classify the GO category. Adjusted P-values were computed for the GO terms of all target genes by Multiple Test Adjustment of BH. And GO terms with an adjusted *p*-value < 0.05 were selected. Based on the interactions of miRNAs and target genes in the Sanger miRNA database, the miRNA function network was built. In addition, for identifying the biological pathways associated with recurrence of HCC, we also performed the KEGG enrichment analysis of target genes.

## 4. Conclusions

By analyzing miRNA expression profiling, we identified 32 miRNAs that are differentially expressed among samples of HCC patients with early and late recurrence and validated that their expression patterns could be useful biomarkers to distinguish these two different types of HCC recurrence. Furthermore, we shed new light on links between miRNA-regulated genes controlling pivotal cancer-related pathways and HCC recurrence. Our data not only suggest valuable biomarkers for diagnosis but also provide putative mechanistic insights into molecular control of HCC recurrence, and this would ultimately improve the treatment stratifications of HCC patients.

## Figures and Tables

**Table 1:** Clinicopathological characteristics of HCC patients.

Clinical Features	Early recurrent cohort (*n* = 8)	Late recurrent cohort (*n* = 7)	*p*-Value
Age (year)			
mean	53.5 ± 7.3	65.0 ± 6.6	0.007 a
range			

Gender (num)			
Male	8	6	0.268 b
Female	0	1	

Tumor size(cm)			
≤5	5	6	0.31 b
>5	3	1	

Child-Pugh class			
A	8	3	0.013 b
B	0	4	

Cirrhosis			
yes	7	5	0.438 b
no	1	2	

Tumor grade (pathology)			
High	3	2	0.218 c
Medium	5	3	
Low	0	2	

HBV			
(+)	7	6	0.919 b
(−)	1	1	

Multinodular			
yes	5	4	0.833 b
no	3	3	

Micro-vascular invasion			
yes	4	7	0.029 b
no	4	0	

Serum AFP level (ng/mL)	1336 (18.5, 71775)	135 (2.35, 591.8)	0.029 b

Mean recurrence-free survival	2.27 ± 1.69	44.76 ± 21.91	0.000 a

AFP: alpha-fetoprotein

a *t*-test

b Chi-square test

c Mann-Whitney U Test

**Table 2:** Summary of differentially expressed miRNAs.

miRNA ID	Chromosome coordinates	Score(d)	Fold Change	Status
hsa-miR-636	chr17: 74732532-74732630 [−]	0.773301691	28.96554407	up
hsa-miR-671	chr7: 150935507-150935624 [+]	0.617872345	27.76690648	up
hsa-miR-489	chr7: 93113248-93113331 [−]	0.8255668	17.98873011	up
hsa-miR-26a	chr3: 38010895-38010971 [+]	0.473722595	12.24271937	up
hsa-miR-320	chr8: 22102475-22102556 [−]	0.67291134	9.39225375	up
hsa-miR-628	chr15: 55665138-55665232 [−]	0.814261793	7.507041018	up
hsa-miR-505	chrX: 139006307-139006390 [−]	0.64190506	7.495093696	up
hsa-miR-100	chr11: 122022937-122023016 [−]	0.424532207	7.089941626	up
hsa-miR-664	chr1: 220373880-220373961 [−]	0.950787576	6.755908146	up
hsa-miR-942	chr1: 117637265-117637350 [+]	0.525349883	6.459339163	up
hsa-miR-192	chr11: 64658609-64658718 [−]	0.31306747	6.351855536	up
hsa-miR-339	chr7: 1062569-1062662 [−]	0.665577861	4.870026318	up
hsa-miR-29c	chr1: 207975197-207975284 [−]	0.458932548	4.169859654	up
hsa-miR-191	chr3: 49058051-49058142 [−]	0.385561147	3.935817957	up
hsa-miR-196b	chr7: 27209099-27209182 [−]	0.44164008	3.877894721	up
hsa-miR-190b	chr1: 154166141-154166219 [−]	0.500700227	2.874169033	up
hsa-miR-18b	chrX: 133304071-133304141 [−]	−0.057442249	−2.12894251	down
hsa-miR-106b	chr7: 99691616-99691697 [−]	−0.506823027	−2.190671054	down
hsa-miR-92a	chr13: 92003568-92003645 [+]	−0.528714339	−2.351269626	down
hsa-miR-186	chr1: 71533314-71533399 [−]	−0.59646555	−2.658452545	down
hsa-miR-204	chr9: 73424891-73425000 [−]	−0.48889237	−2.960625198	down
hsa-miR-1180	chr17: 19247819-19247887 [−]	−0.699507876	−3.031757322	down
hsa-miR-193a-5p	chr17: 29887015-29887102 [+]	−0.811044599	−3.042132652	down
hsa-miR-23b	chr9: 97847490-97847586 [+]	−0.726168543	−3.324757638	down
hsa-miR-376c	chr14: 101506027-101506092 [+]	−0.78522436	−3.845047161	down
hsa-miR-15a	chr3: 160122376-160122473 [+]	−1.704080648	−3.895068364	down
hsa-miR-769	chr19: 46522190-46522307 [+]	−0.860296951	−4.785376714	down
hsa-miR-99b	chr19: 52195865-52195934 [+]	−1.068532726	−5.370680926	down
hsa-miR-125b	chr11: 121970465-121970552 [−]	−0.571324674	−6.378593023	down
hsa-miR-10b	chr2: 177015031-177015140 [+]	−0.49959367	−6.668372154	down
hsa-miR-30b	chr8: 135812763-135812850 [−]	−0.474034967	−11.45095534	down
hsa-miR-145	chr5: 148810209-148810296 [+]	−0.601999659	−23.69139215	down

**Table 3:** This table lists the enriched KEGG pathways, number of differentially expressed genes in a specific pathway, enriched gene ID and adjusted *p*-value by the hyper-geometric test.

KEGG pathway	Number of genes	Entrez gene ID	*p*-Value
Focal adhesion	15	1284 331 5578 5156 7057 3725 595 7791 5880 1398 10000 896 5062 80310 2932	0.0000616
Pathways in cancer	13	2246 2261 5925 5728 8322 2475 8325 1871 3845 6932 1857 867 7423	0.0001
MAPK signaling pathway	9	2246 2261 6195 23162 4296 2872 3845 3552 6197	0.0002
Bladder cancer	5	3845 2261 5925 7423 1871	0.0003
Prostate cancer	6	3845 6932 5925 5728 2475 1871	0.0005
mTOR signaling pathway	5	6198 2475 6195 6197 7423	0.0005
Tight junction	7	3845 9223 71 2771 5728 5580 2770	0.0007
Wnt signaling pathway	7	56998 6932 5529 1857 8322 8325 8945	0.0011
Colorectal cancer	5	3845 6932 1857 8322 8325	0.0021
Gap junction	5	3845 2771 1453 2770 107	0.0027
Basal cell carcinoma	4	6932 1857 8322 8325	0.0027
Long-term potentiation	4	3845 6195 6197 107	0.0052
Pancreatic cancer	4	3845 5925 7423 1871	0.0054
ErbB signaling pathway	4	3845 6198 867 2475	0.0099
Fc gamma R-mediated phagocytosis	4	6198 382 10092 5580	0.0137
Leukocyte transendothelial migration	4	71 4478 2771 2770	0.0254
Neurotrophin signaling pathway	4	3845 6195 6197 5580	0.0302
